# 2097 Johns Hopkins School of Medicine ClinicalTrials.gov Program challenges and successes

**DOI:** 10.1017/cts.2018.291

**Published:** 2018-11-21

**Authors:** Anthony Keyes, Nidhi M. Atri, Prince S. Nuamah

**Affiliations:** Johns Hopkins University School of Medicine

## Abstract

OBJECTIVES/SPECIFIC AIMS: Educate the general public, investigators, and institutional leadership on the importance of clinical trial registration and results reporting. Share success as a means to develop national best practices. METHODS/STUDY POPULATION: Developed a Project Charter; Spoke to several peer institutions; Update institutional policy. RESULTS/ANTICIPATED RESULTS: Since launching the Program in June 2016, the number of records submitted to ClinicalTrials.gov has increased 14% (852–971). At the same time, compliance with late results has increased by over 92% (111–9). DISCUSSION/SIGNIFICANCE OF IMPACT: Clinical Trial registration and results reporting is sub-par at many institutions. We have established a successful program that others can emulate. Institutions can increase transparency of clinical trials as well as prevent civil monetary penalties ($11,569/d/study) and loss of grant funding.

